# Viscometric Studies in Dilute Solution Mixtures of Chitosan and Microcrystalline Chitosan with Poly(vinyl alcohol)

**DOI:** 10.1007/s10953-013-0053-3

**Published:** 2013-08-15

**Authors:** Katarzyna Lewandowska

**Affiliations:** Faculty of Chemistry, Chair of Chemistry and Photochemistry of Polymers, Nicolaus Copernicus University, 7 Gagarin Street, 87-100 Toruń, Poland

**Keywords:** Chitosan, Polymer blends, Miscibility, Poly(vinyl alcohol), Interaction parameter, Miscibility criteria, Intrinsic viscosity

## Abstract

The viscosity behavior of aqueous mixtures formed by a polyelectrolyte (A) and a neutral polymer (B), such as chitosan/poly(vinyl alcohol) (Ch/PVA) and microcrystalline chitosan/poly(vinyl alcohol) (MCCh/PVA), have been investigated at 25 °C. The intrinsic viscosity and the viscosity interaction parameter of each polymer in 0.1 mol·dm^−3^ CH_3_COOH/0.2 mol·dm^−3^ NaCl solution as well as the ternary systems (polymer A/polymer B/solvent) have been determined and have served for estimation of the miscibility of different polymer mixtures by means of the method of classical dilution. By comparing the experimental and ideal viscosity data it is clearly seen that the satisfaction of the miscibility criterion depends on the definition of the ideal parameter $b_{\text{m}}^{\text{id}}$. If the $b_{\text{m}}^{\text{id}}$ parameter is defined according to the Krigbaum–Wall criterion and Garcia criterion, the investigated blends of Ch/PVA satisfy the miscibility criterion. In the case of MCCh/PVA blends, the polymeric components show poor miscibility. Additionally, the viscosity results show that the degree of miscibility depends on the molecular weight of chitosan and on the degree of PVA hydrolysis.

**Electronic supplementary material:**

The online version of this article (doi:10.1007/s10953-013-0053-3) contains supplementary material, which is available to authorized users.

## Introduction

The physico-chemical properties of high molecular weight compounds can be modified by various methods, one of them is simple blending with other macromolecular compounds, which assures the required useful behavior in manufacturing and applications. In this study a natural polysaccharide, chitosan (Ch), has been blended with poly(vinyl alcohol) (PVA). Ch is a modified natural polymer, that is non-toxic, bioactive and biodegradable, derived by deacetylation of chitin. It is the second most widespread biopolymer in nature after cellulose [1, 2]. This biopolymer is a weak polybase, showing a polyelectrolytic effect in aqueous dilute acidic solution. Specific properties of Ch have resulted in an increasing interest in its investigation and application, e.g. in medicine, pharmacy and the food and cosmetic industries [3, 4]. The growth in practical applications for this natural polymer is related to the modification of its properties through suitable changes of molecular, supermolecular and chemical structure. Microcrystalline chitosan (MCCh) is a special form of Ch, which is prepared via its physicochemical modification using aqueous hydroxides and their salts [5, 6]. A previous study [7] showed that the thermal properties of films produced by blending Ch and PVA depend on the degree of hydrolysis of PVA and on the molecular weight of Ch. To my knowledge the physico-chemical properties of MCCh with PVA mixtures have not been investigated before. In the present work, the miscibility of Ch/PVA and MCCh/PVA have been studied by viscometry. One of the procedures used for the estimation of miscibility in dilute solution is the determination of viscosity interaction parameters for mixtures of polymers in different proportions. This method is often used because of its simplicity and low cost [8–13].

In the method of classical dilution, the miscibility is estimated by comparison of the experimental and ideal values of $b_{\text{m}}^{\text{exp}}$ and $\left[ \eta \right]_{\text{m}}^{\exp }$. The values of $b_{\text{m}}^{\text{exp}}$ and $[\eta ]_{\text{m}}^{\exp }$ are determined from a plot of reduced viscosity versus the mixture concentrations {*η*_sp_/*c* = *f*(*c*)} (Eq. 1) for solutions containing both polymers:

1 $$ \frac{{(\eta_{\text{sp}} )_{\text{m}} }}{{c{}_{\text{m}}}} = \left[ \eta \right]_{\text{m}} + b_{\text{m}} c_{\text{m}} $$

where (*η*_sp_)_m_ is the specific viscosity of the polymer mixture, $b_{\text{m}}^{\text{exp}}$ the experimental value of the viscosity interaction parameter of the polymer mixture, $\left[ \eta \right]_{\text{m}}^{\exp }$ the experimental value of intrinsic viscosity of the polymer mixture, $[\eta ]_{\text{m}}^{{}}$ the intrinsic viscosity of the polymer mixture and *c*_m_ the total concentration of the mixture.

The viscosity interaction parameter *b* is related to Huggins coefficient *k* by the equation:

2 $$ b\, = \,k\,\left[ \eta \right]^{2} $$

Krigbaum and Wall [8] have defined the ideal value of the interaction parameter $b_{\text{m}}^{\text{id}}$ by the expression:

3 $$ b_{\text{m}}^{\text{id}} = b_{\text{AA}} w_{\text{A}}^{2} + b_{\text{BB}}^{{}} w_{\text{B}}^{ 2} + 2b_{\text{AB}}^{\text{id}} w_{\text{A}} w_{\text{B}} $$

4 $$ b_{\text{AB}}^{\text{id}} = b_{\text{AA}}^{{{1 \mathord{\left/ {\vphantom {1 2}} \right. \kern-0pt} 2}}} b_{\text{BB}}^{{{1 \mathord{\left/ {\vphantom {1 2}} \right. \kern-0pt} 2}}} $$

where: *b*_AA_, *b*_BB_, *b*_AB_ are the interaction parameters of like (AA, BB) and unlike (AB) polymer molecules, respectively, and *w*_A_ and *w*_B_ are the weight fractions of polymers A and B, respectively.

The polymer blend is miscible if $\Updelta b_{\text{m}} = b_{\text{m}}^{ \exp } - b_{\text{m}}^{\text{id}} > 0$ and immiscible if $\Updelta b_{\text{m}} = b_{\text{m}}^{ \exp } - b_{\text{m}}^{\text{id}} < 0$.

Garcia et al. [14] defined the ideal value of the interaction parameter, $b_{\text{m}}^{\text{id}}$, as:

5 $$ b_{\text{m}}^{\text{id}} = b_{\text{AA}} w_{\text{A}}^{ 2} + b_{\text{BB}} w_{\text{B}}^{ 2} $$

Additionally, Garcia et al. [14] have proposed another miscibility criterion, which is based on the difference between the experimental and ideal values of [*η*]_m_. If $\Updelta \left[ \eta \right]_{\text{m}} = \left[ \eta \right]_{\text{m}}^{ \exp } - \left[ \eta \right]_{\text{m}}^{\text{id}} \,\, < \,\,0$ the system is miscible, and if $\Updelta \left[ \eta \right]_{\text{m}} = \left[ \eta \right]_{\text{m}}^{ \exp } - \left[ \eta \right]_{\text{m}}^{\text{id}} \,\, > \,\,0$ the system is immiscible. The value of $\left[ \eta \right]_{\text{m}}^{\text{exp}}$ is determined from the intercept of the plot according to Eq. 1, whereas $\left[ \eta \right]_{\text{m}}^{\text{id}}$ is obtained from Eq. 5. The values of [*η*]_A_ and [*η*]_B_ are given in Table 1.

6 $$ \left[ \eta \right]_{\text{m}}^{\text{id}} = \left[ \eta \right]_{\text{A}} w_{\text{A}} + \left[ \eta \right]_{\text{B}} w_{\text{B}} $$

**Table 1:** Characteristics of the polymer samples

Polymer	*a*	*K* × 10^4^ (dL·g^−1^)	[*η*] (dL·g^−1^)	*T* (K)	$M_{\text{v}}$ × 10^−3^ (g·mol^−1^)	DD (%)	DH (%)	Source
Ch I	0.93	0.181^a^	3.12	298	427	83		Institute of Sea Fisher (Poland)
Ch II	0.93	0.181^a^	9.35	298	1390	79		Aldrich
MCCh	0.93	0.181^a^	7.26	298	1059	84		Our laboratory
PVA88	0.58	8.0^b^	0.76	298	136		88	Loba
PVA99	0.61	6.9^b^	0.82	298	110		99	Aldrich

*a*, *K* are the parameters of Mark-Houwink-Sakurada equation, the *M*_v_ is the viscosity average molecular weight, *DD* is the degree of deacetylation of chitosan, *DH* is the degree of hydrolysis of PVA

^a^Solvent: 0.1 mol·dm^−3^ CH_3_COOH, 0.2 mol·dm^−3^ NaCl [15]

^b^Solvent: water [16]

The aim of this study is to evaluate the miscibility of Ch with PVA and MCCh with PVA on the basis of experimental and ideal values of the viscosity interaction parameters: *b*_m_ and [*η*]_m_, which were calculated from viscosity measurements of dilute solutions. The determination of the miscibility degree of the above polymer pairs has been done by means of two criteria. The influence of molecular weight of Ch and the degree of PVA hydrolysis on the hydrodynamic properties of blend solutions have also been investigated.

## Experimental

### Materials

The physico-chemical characteristics of Ch and PVA samples are given in Table 1. Distilled water and 0.1 mol·dm^−3^ CH_3_COOH/0.2 mol·dm^−3^ NaCl were used as solvent. The pH = 3.25 at 25 °C for the polymer solvent. Sodium chloride and acetic acid (analytical reagent grade) were obtained from POCh, Poland.

The viscosity average molecular weight $M_{\text{v}}$ of Ch and PVA was measured with an Ubbelohde viscometer and calculated from the viscosities of the solutions according to the Mark-Houwink-Sakurada equation [17, 18]:

7 $$ \left[ \eta \right] = K\bar{M}_{\text{v}}^{\text{a}} $$

The temperature of the viscosity measurement, the solvent and the Mark-Houwink-Sakurada constants *K* and *a* for evaluating $M_{\text{v}}$ are specified in Table 1.

The degree of deacetylation (DD) of Ch was estimated according to Polish Standards (PN-87/A-86850).

The degree of hydrolysis (DH) of PVA refers to the content of residual vinyl acetate groups. It was determined titrimetrically according to the Japan Industrial Standard [19].

### Preparations

Microcrystalline chitosan was prepared in our laboratory from chitosan (Ch II) by means of the original method described in the literature [5]. To the reactor containing 1 % Ch solution in 1 % aqueous acetic acid was slowly dropped 2 % aqueous sodium hydroxide solution with intensive stirring at room temperature. The subsequent procedure was the same as described earlier [5]. Ch, MCCh and PVA were solubilized, separately, in aqueous 0.1 mol·dm^−3^ CH_3_COOH/0.2 mol·dm^−3^ NaCl solution and then mixed in different proportions.

### Viscosity Measurements

Viscosity measurements of dilute polymer solution (c < 0.2 %) were carried out in a controlled thermostatic bath at 25 ± 0.1 °C using an Ubbelohde capillary viscometer with a viscometer constant of 11.75 s^2^. Distilled water was used as the calibration fluid for the Ubbelohde viscometer for temperatures in the range 18–30 °C. The flow times were recorded with an accuracy of ±0.01 s. The flow time of each solution was determined as the average of several readings. Before measurement, the solutions were filtered through G1 sintered glass filters. The intrinsic viscosity [*η*], the interaction parameter *b* and Huggins coefficient *k* values were determined according to the Heller [20] procedure from data obtained for solutions at 5 concentrations. Kinetic energy corrections were taken into account for the evaluation of the intrinsic viscosity, which was determined by extrapolation to infinite dilution (zero solute concentration).

## Results and Discussion

**Fig. 1:**
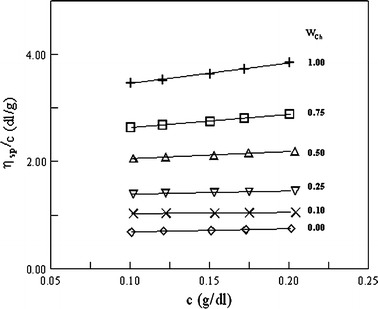
The reduced viscosity versus polymer concentration for Ch I, PVA(88) and their blends in 0.1 mol·dm^−3^ CH_3_COOH + 0.2 mol·dm^−3^ NaCl at 25 °C; w_Ch_ is the weight fraction of chitosan

**Fig. 2:**
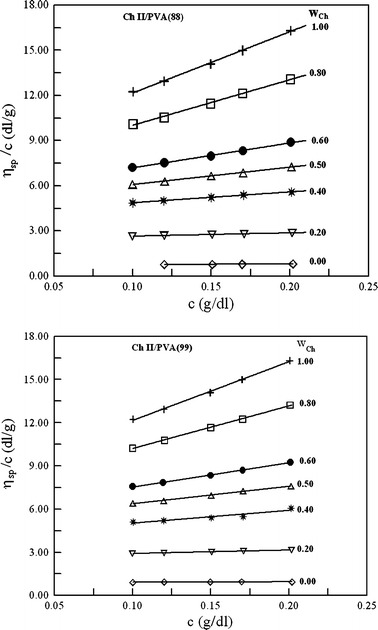
The reduced viscosity versus polymer concentration for Ch II, PVA [PVA(88) and PVA(99)] and their blends in 0.1 mol·dm^−3^ CH_3_COOH + 0.2 mol·dm^−3^ NaCl at 25 °C; w_Ch_ is the weight fraction of chitosan

**Fig. 3:**
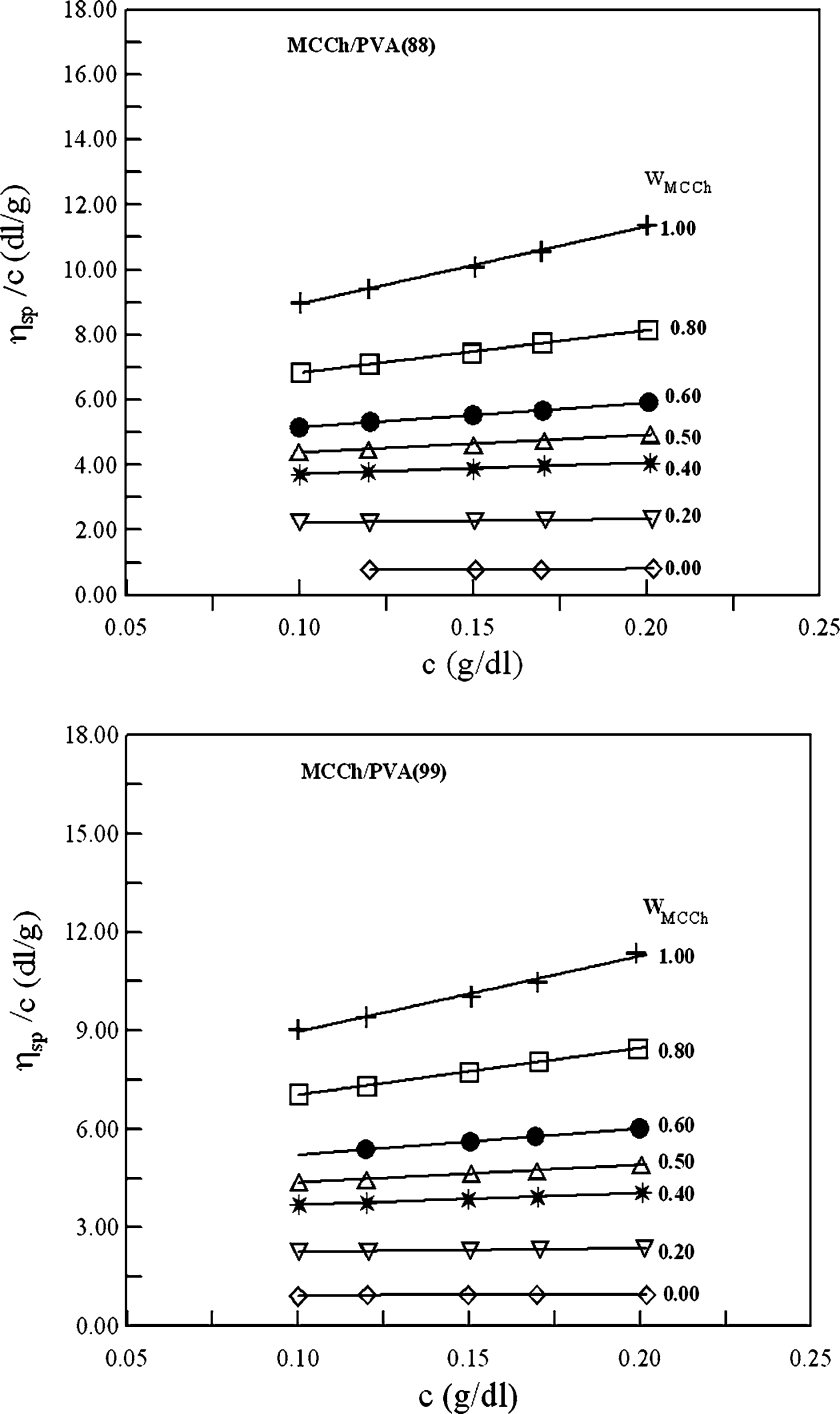
The reduced viscosity versus polymer concentration for MCCh, PVA [PVA(88) and PVA(99)] and their blends in 0.1 mol·dm^−3^ CH_3_COOH + 0.2 mol·dm^−3^ NaCl at 25 °C; w_MCCh_ is the weight fraction of microcrystalline chitosan

Figures 1, 2 and 3 show the plots of reduced viscosity versus the concentration for Ch, PVA, MCCh and their blends. All the plots show linear behavior in the range of concentration studied, indicating that the intrinsic viscosity can be determined by linear extrapolation to zero concentration. Ch is a cationic polyelectrolyte and, in aqueous acetic acid solution, in the limit of low ionic strength the electrostatic interactions are weakly screened. Hence, the polyelectrolytic effect of Ch is observed [21, 22]. In this study, the ionic strength in the investigated system was high enough to prevent the Ch chains showing a polyelectrolyte effect.

The plots of *η*_sp_/*c* versus *c* for Ch/PVA and MCCh/PVA blends lies between the plots for the starting polymers. Thus the intermolecular interaction between Ch/PVA or MCCh/PVA is not very strong [8, 9, 13, 23]. In Tables 2 and 3, the values of experimental and ideal intrinsic viscosities are collected for the investigated systems. The $\left[ \eta \right]_{\text{m}}^{\exp }$ values were obtained according to Heller’s procedure [20], which was developed from the inverse of the Huggins and Kraemer equations. This method gives some advantages, including high accuracy of determination of the intrinsic viscosity and slope constants because the slopes of the plots are smaller compared to the slopes of Huggins and Kraemer plots [11, 18, 20].

**Table 2:** A comparison of the experimental and ideal viscometric parameters of the Ch + PVA blends and dependence on the assumed definition of the ideal values of these parameters

*w*_ch_	$[\eta ]_{\text{m}}^{\exp }$ (dL·g^−1^)	$\left[ \eta \right]_{\text{m}}^{\text{id}}$ (dL·g^−1^)	$\Updelta \left[ \eta \right]$	$b_{\text{m}}^{\exp }$ (dL·g^−1^)^2^	$b_{\text{m}}^{{{\text{id}}^{ * } }}$ (dL·g^−1^)^2^	$\Updelta b_{\text{m}}$	$b_{\text{m}}^{{{\text{id}}^{ * * } }}$ (dL·g^−1^)^2^	$\Updelta b_{\text{m}}$
Ch I/PVA(88)								
0.10	0.99 ± 0.02	0.92 ± 0.02	0.07	0.30 ± 0.040	0.37 ± 0.04	−0.07	0.22 ± 0.031	0.08
0.25	1.33 ± 0.02	1.28 ± 0.02	0.05	0.67 ± 0.095	0.65 ± 0.09	0.02	0.33 ± 0.046	0.34
0.50	1.92 ± 0.03	1.90 ± 0.03	0.03	1.30 ± 0.180	1.30 ± 0.18	0.00	0.84 ± 0.120	0.46
0.75	2.41 ± 0.04	2.51 ± 0.05	−0.09	2.10 ± 0.300	2.10 ± 0.30	0.00	1.80 ± 0.300	0.30
Ch II/PVA(88)								
0.20	2.45 ± 0.02	2.48 ± 0.02	−0.03	1.85 ± 0.26	1.79 ± 0.25	0.06	1.12 ± 0.16	0.73
0.40	4.26 ± 0.04	4.19 ± 0.05	0.07	5.80 ± 0.81	5.12 ± 0.72	0.68	4.12 ± 0.58	1.68
0.50	5.16 ± 0.04	5.05 ± 0.05	0.11	8.50 ± 1.20	7.42 ± 1.04	1.08	6.38 ± 0.89	2.12
0.60	5.96 ± 0.06	5.91 ± 0.06	0.05	11.4 ± 1.60	10.2 ± 1.43	1.20	9.15 ± 1.28	2.25
0.80	7.88 ± 0.07	7.63 ± 0.08	0.25	19.9 ± 2.79	16.9 ± 2.37	3.00	16.23 ± 2.27	3.67
Ch II/PVA(99)								
0.20	2.69 ± 0.03	2.58 ± 0.03	0.11	2.16 ± 0.29	2.14 ± 0.29	0.02	1.22 ± 0.17	0.95
0.40	4.33 ± 0.13	4.27 ± 0.06	0.06	6.38 ± 0.89	5.55 ± 0.78	0.83	4.17 ± 0.58	1.38
0.50	5.40 ± 0.06	5.12 ± 0.07	0.28	9.05 ± 1.27	7.85 ± 1.10	1.20	6.42 ± 0.90	1.43
0.60	6.24 ± 0.06	6.00 ± 0.06	0.24	11.68 ± 1.64	10.56 ± 1.48	1.12	9.18 ± 1.29	2.50
0.80	8.08 ± 0.08	7.66 ± 0.10	0.42	18.94 ± 2.65	17.16 ± 2.40	1.78	16.24 ± 2.27	0.92

Solvent: 0.1 mol·dm^−3^ CH_3_COOH + 0.2 mol·dm^−3^ NaCl. $b_{\text{m}}^{{{\text{id}}^{ * } }}$ determined according to Krigbaum and Wall [8], $b_{\text{m}}^{{{\text{id}}^{ * * } }}$ determined according to Garcia et al. [14]

*w*_*Ch*_ the weight fraction of chitosan

**Table 3:** A comparison of the experimental and ideal viscometric parameters of the MCCh + PVA blends and dependence on the assumed definition of the ideal values of these parameters

*w*_MCCh_	$[\eta ]_{\text{m}}^{\exp }$ (dL·g^−1^)	$\left[ \eta \right]_{\text{m}}^{\text{id}}$ (dL·g^−1^)	$\Updelta \left[ \eta \right]$	$b_{\text{m}}^{\exp }$ (dL·g^−1^)^2^	$b_{\text{m}}^{{{\text{id}}^{ * } }}$ (dL·g^−1^)^2^	$\Updelta b_{\text{m}}$	$b_{\text{m}}^{{{\text{id}}^{ * * } }}$ (dL·g^−1^)^2^	$\Updelta b_{\text{m}}$
MCCh/PVA(88)								
0.20	2.11 ± 0.03	2.05 ± 0.03	0.06	1.03 ± 0.14	1.26 ± 0.18	−0.23	0.73 ± 0.10	0.30
0.40	3.39 ± 0.03	3.34 ± 0.04	0.05	3.10 ± 0.43	3.34 ± 0.47	−0.24	2.56 ± 0.36	0.54
0.50	3.91 ± 0.04	3.98 ± 0.04	−0.07	4.43 ± 0.62	4.76 ± 0.67	−0.33	3.94 ± 0.55	0.49
0.60	4.51 ± 0.04	4.63 ± 0.05	−0.12	5.89 ± 0.82	6.43 ± 0.90	−0.54	5.64 ± 0.79	0.25
0.80	5.78 ± 0.06	5.92 ± 0.06	−0.14	9.69 ± 1.36	10.25 ± 1.44	−0.56	9.98 ± 1.40	−0.29
MCCh/PVA(99)								
0.20	2.15 ± 0.02	2.16 ± 0.02	−0.01	0.97 ± 0.14	1.57 ± 0.22	−0.60	0.84 ± 0.12	0.13
0.40	3.35 ± 0.03	3.44 ± 0.04	−0.09	3.14 ± 0.44	3.73 ± 0.52	−0.59	2.65 ± 0.37	0.49
0.50	3.90 ± 0.04	4.08 ± 0.04	−0.18	4.41 ± 0.62	5.17 ± 0.72	−0.76	4.03 ± 0.56	0.38
0.60	4.56 ± 0.04	4.71 ± 0.05	−0.15	6.24 ± 0.87	6.83 ± 0.96	−0.59	5.74 ± 0.80	0.50
0.80	5.91 ± 0.06	5.99 ± 0.06	−0.08	10.46 ± 1.46	10.87 ± 1.52	−0.41	10.13 ± 1.42	0.33

Solvent: 0.1 mol·dm^−3^ CH_3_COOH + 0.2 mol·dm^−3^ NaCl. $b_{\text{m}}^{{{\text{id}}^{ * } }}$ determined according to Krigbaum and Wall [8], $b_{\text{m}}^{{{\text{id}}^{ * * } }}$ determined according to Garcia et al. [14]

The [*η*]^id^ values were calculated by using Eq. 6. As is seen from Table 2 for Ch I + PVA(88) and Ch II + PVA(88) blends with *w*_Ch_ ≤ 0.6, the values of ∆[*η*] are low and marginally exceeded the range of the experimental error. Greater differences in the values of ∆[*η*] are observed for Ch II + PVA(99) and Ch II + PVA(88) blends with *w*_Ch_ = 0.8 (*w*_Ch_ is the weight fraction of Ch in the blend). This indicates that the influence of the degree of hydrolysis of PVA on the miscibility of polymeric component is more pronounced than the molecular weight of Ch. Table 2 also shows a comparison of the experimental $b_{\text{m}}^{\exp } \,$ and the ideal $b_{\text{m}}^{\text{id}} \,$ viscometric parameters. The ideal values are based on two equations proposed by Krigbaum and Wall [8] (Eq. 3) and Garcia [14] (Eq. 5). For the miscibility criterion of Krigbaum and Wall [8], the ∆*b*_m_ values are positive or zero within the experimental error. In the case of Garcia’s criterion [14], the values of parameter Δ*b*_m_ are also positive for all of the investigated systems, exceeding distinctly the range of the experimental error, with the exception of the blend for low content of PVA (w_PVA_ ≤ 0.25) in Ch I + PVA(88) and Ch II + PVA(99) blends. So, the Ch + PVA blends satisfy criteria proposed by Krigbaum and Wall [8] and Garcia et al. [14]. This conclusion partially confirmed earlier studies in the solid state [7] where PVA(88) shows partial miscibility with Ch I. The studies in the solid state were carried out for mixtures obtained from aqueous acetic acid solutions. This may affect the observed differences. It is known that the solvent plays an important role in the miscibility of polymeric compounds [12, 14, 24]. A reasonable interpretation is that in different solvents, the interactions between two different polymers are quite different.

The parameters of the two different miscibility criteria are summarized in Table 3 for MCCh + PVA blends. As can be seen, in the case of the Krigbaum and Wall [8] criterion, the ∆*b*_m_ values are negative for all investigated systems, exceeding the range of the experimental error. Thus, these blends are poorly miscible in solution in comparison with Ch/PVA blends. This is also confirmed by the criterion of Garcia et al. [14], in which the parameters ∆*b*_m_ values are positive but the values are much smaller than for Ch/PVA blends. For MCCh/PVA blends with *w*_PVA_ ≤ 0.5, the ∆*b*_m_ values do not indicate deviations beyond the range of experimental error.

## Conclusions

In this paper, the miscibility of dilute aqueous Ch I + PVA, Ch II + PVA and MCCh + PVA blend solution are investigated. Two different criteria to estimate the polymer–polymer miscibility in dilute solution, the Krigbaum and Wall criterion and Garcia et al. criteria, are considered. As is known, the satisfaction of the miscibility criterion depends on the definition of the ideal parameter values [11, 14]. If the ideal $b_{\text{m}}^{\text{id}} \,$ viscometric parameter is defined according to Garcia et al. and Krigbaum and Wall, then the investigated blends of Ch + PVA blends satisfy the miscibility criterion. Thus, the PVA sample shows miscibility with Ch at *w*_Ch_ > 0.2. In the case of MCCh + PVA blends, only the Garcia et al. criterion is satisfied. This behavior may indicate a poorer miscibility of the components and stronger repulsive interactions.

## Electronic supplementary material

Below is the link to the electronic supplementary material.

Supplementary material 1 (DOC 34 kb)
