# Supplementary material for: Viscometric Studies in Dilute Solution Mixtures of Chitosan and Microcrystalline Chitosan with Poly(vinyl alcohol)
Source: J Solution Chem. 2013 Aug 15;42(8):1654–62. doi: 10.1007/s10953-013-0053-3 (PMC3769582; doi:10.1007/s10953-013-0053-3)
Supplement: Supplementary file 1 — Supplementary material 1 (DOC 34 kb) [file 10953_2013_53_MOESM1_ESM.doc]

**Supplementary Material for**

**Viscometric Studies in Dilute Solution Mixtures of Chitosan and Microcrystalline Chitosan with Poly(Vinyl Alcohol)**

**Katarzyna Lewandowska**

**Table 1** The experimental values of the intrinsic viscosity and Huggins coefficient for polymers and mixtures

| Ch I + PVA(88) Ch II + PVA(88) Ch II + PVA(99)  *w*_Ch_ [*η*] *k*_H_ [*η*] *k*_H_ [*η*] *k*_H_  (cm^3^·g^−1^) (cm^3^·g^−1^) (cm^3^·g^−1^) |
| --- |
| 0.0 67.0 0.52 76 0.30 89 0.41  0.1 99.1 0.30  0.2 244.6 0.31 268.6 0.30  0.25 133.0 0.38  0.4 425.8 0.31 433.1 0.34  0.5 192.2 0.36 515.5 0.32 540.2 0.31  0.6 595.7 0.32 624.0 0.30  0.75 241.2 0.36  0.8 788.0 0.32 808.1 0.29  1.0 312.4 0.32 934.9 0.29 934.9 0.29 |

**Table 2** The experimental values of the intrinsic viscosity and Huggins coefficient for polymers and mixtures

| MCCh /PVA(88) MCCh /PVA(88)  *w*_Ch_ [*η*] *k*_H_ [*η*] *k*_H_  (cm^3^·g^−1^) (cm^3^·g^−1^) |
| --- |
| 0.0 76.0 0.30 89 0.41  0.2 211.3 0.23 215.2 0.21  0.4 339 0.27 335.0 0.28  0.5 390.7 0.29 390.1 0.29  0.6 450.8 0.29 455.9 0.30  0.8 578.0 0.29 590.6 0.30  1.0 720.9 0.30 720.9 0.30 |
